# Paratesticular Pleomorphic Rhabdomyosarcoma: A Report of Two Cases

**DOI:** 10.1155/2013/807979

**Published:** 2013-02-12

**Authors:** Rami Boulma, Mohamed Mourad Gargouri, Ahmed Sallemi, Mohamed Chlif, Zouhaier Fitouri, Yosri Kallel, Yassine Nouira

**Affiliations:** Department of Urology, La Rabta University Hospital, 1007 Tunis, Tunisia

## Abstract

Pleomorphic rhabdomyosarcoma (RMS) is a rare tumor with an aggressive behavior, described mainly in adulthood. Herein we present two cases of paratesticular pleomorphic RMS in 71- and 16-year-old patients with metastases at initial diagnosis. Histological, immunohistochemical, and ultrastructural findings were essential to confirm diagnosis. Few months after radical orchiectomy, both patients died before or just after starting adjuvant chemotherapy.

## 1. Introduction

Rhabdomyosarcoma (RMS) is a highly malignant mesenchymal tumor thought to originate from immature striated muscle. It is characterized by the presence of cells having an identifiable striated muscular differentiation with rhabdomyoblasts cells [1]. Twenty percent of all cases of RMS arise from the genitourinary system [2]. The pleomorphic form is typically more frequent in adults and has a poor prognosis [1].

Herein, we report two cases of pleomorphic RMS diagnosed in an adolescent and adult patient with multiple metastases at initial diagnosis.

## 2. Case N 1

**Figure 1:**
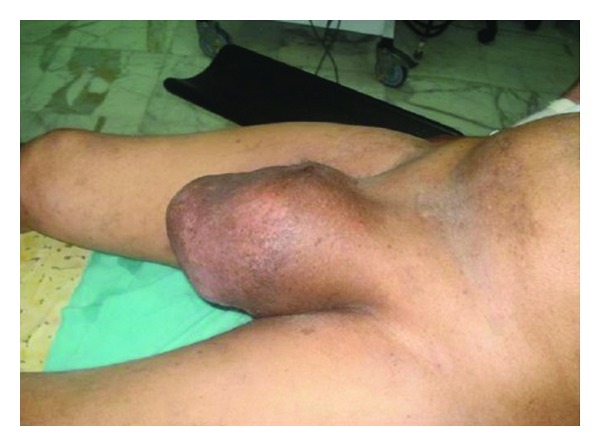
Physical exam: scrotal left mass measuring 20 cm.

A 71-year-old man presented to our urology department for a 2-year history of testicular swelling and a painless mass in the left scrotum. The patient felt uncomfortable with that mass with a feeling of heaviness. His past medical history was unremarkable. The physical examination showed a 20 cm solid mass occupying the left scrotal region with a buried penis (Figure 1).

**Figure 2:**
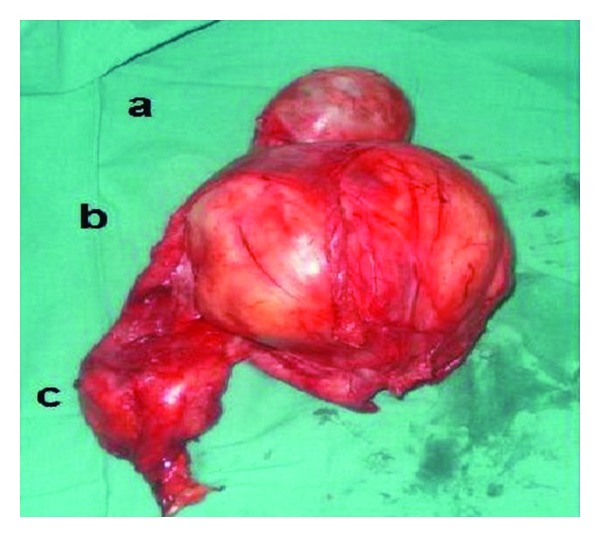
Macroscopic findings: (a) normal testis, para testicular tumors measuring 15 cm (b) and 4 cm (c).

The mass seemed to be independent from the testis and ultrasonography confirmed that it was an extratesticular solid mass. A contrast enhanced computed tomography (CT) revealed multiple pulmonary lesions and retroperitoneal lymph nodes. The diagnosis of paratesticular tumour with multiple metastases was made and surgical excision with adjuvant chemotherapy was decided. The patient underwent left high inguinal orchiectomy. The testis was macroscopically normal and two tumours of 15 and 4 cm were localized in paratesticular region (Figure 2). The total weight of specimen was 1.2 Kg. On macroscopic examination, a white, encapsulated partially calcified tumour was observed. Microscopic histopathologic examination revealed a malignant mesenchymatous proliferation made of pleomorphic cells, arranged in sheets and lobules with cross striations. Mitoses were very numerous. Elsewhere, architecture was made of round, polygonal cells, and large multinucleate cells with strongly eosinophilic and vacuolar cytoplasm and with vesicular chromatin. Large areas of necroses were present. The sarcomatoid proliferation invaded muscular and surrounding fat. The testicular parenchyma was normal. Immunohistochemical study showed that tumour cells were positive for desmin and negative for alpha-smooth-muscle actin. The PS100 showed a positivity of the tumoral cells for desmin, the actin smooth muscle, and myogenin attesting the striated muscular nature with this proliferation. Final pathological diagnosis was paratesticular pleomorphic RMS.

Two months after surgery the patient died from cachexia before starting any adjuvant therapy.

## 3. Case N 2

A 16-year-old boy presented to our department for scrotal swelling evolving for 6 months. He complained of scrotum enlargement and of uncomfortable heavy sensation. He denied any scrotal trauma and his history was unremarkable. Physical examination revealed an enlarged, tender, right scrotum measuring 18 cm with unrecognizable testis.

Ultrasonography revealed that the mass originates from the paratesticular region. CT scan showed a solid process in the right scrotum compressing the two testicles with large retroperitoneal lymph nodes. The diagnosis of paratesticular tumour was considered and a right radical inguinal orchiectomy was performed.

Pathologic microscopic examination revealed a malignant mesenchymal proliferation made out of fusocellular pleomorphic architecture arranged with spindle-shaped cells. Elsewhere, architecture was diffuse; made of polygonal, racket-shaped cells, and large multinucleate cells with strong *hematoxylinophilic eosin *staining, vacuolar cytoplasm, and with ovoid circular core provided with vesicular chromatin. There were also many zones of necroses. Mitoses were very numerous. This sarcomatoid proliferation invaded muscular and fat fabric of vicinity. The testicular parenchyma was normal. The immunohistochemical study using the muscular markers and the PS100 showed a positivity of the tumoral cells for desmin, actin smooth muscle, and myogenin attesting the striated muscular nature with this proliferation. The final pathologic diagnosis was pleomorphic RMS involving paratesticular soft tissues.

An adjuvant chemotherapy protocol using vincristine, actinomycin and ifosfamide was started one month postoperatively and radiotherapy to retroperitoneal lymph nodes was planned thereafter. Unfortunately, the patient died 4 months after surgery.

## 4. Discussion

Malignant tumours of the paratesticular region are uncommon and are mainly sarcomas. The most common histological subtypes of sarcomas are leiomyosarcoma (32%), rhabdo-myosarcoma (RMS, 24%), liposarcoma (20%), and malignant fibrous histiocytoma (MFH, 13%) [3].

RMS is a highly malignant tumour arising from striated muscle cells and is associated with early and wide spread metastasis [1, 3]. Common metastatic sites include lungs, lymph nodes, and bone marrow [4]. The usual sites of origin are the head and neck, extremities, and soft tissues; only 15–20% of the cases arise from the genitourinary tract [1].

RMS is more frequent in males than in females with a ratio of 3 : 1 [5] and occurs mainly in young patients with a mean age of 6 years. There are many subtypes of RMS and all of them may occur in the paratesticular region; however, the commonest subtypes seen are embryonal, alveolar, pleomorphic, and mixed tumours. Pleomorphic RMS is the rarest type and is described mainly in adults and has a poor prognosis, whereas the embryonal type is known to have excellent prognosis [3]. Only spread cases for pleomorphic paratesticular RMS have been reported in adult patients [6–9].

Clinically paratesticular tumour presents as an intrascrotal mass, usually large, sometimes reaching up to the external inguinal ring and compressing the testis and epididymis. In large tumours it may be clinically indistinguishable from testicular tumours. The usual clinical presentation is a painless testicular enlargement. Pain has been reported only in 7% of cases [3], sensation of swelling is more frequent, and a hydrocele can be occasionally present. This could explain why diagnosis can be made late in adults and the tumour could be misdiagnosed as hydrocele; however, ultrasonography can easily demonstrate its solid component and its origin.

Extension of paratesticular tumours to retroperitoneal lymph nodes is common and occurs in up to 30% of patients and lung metastases are present at diagnosis in 14% of cases [10]. Usually a CT is realized to evaluate lymph nodes and distant metastases.

Diagnosis of pleomorphic RMS is histopathological. Diagnosis of this tumour is based on morphologic, immunohistochemical, and ultrastructural findings that identify the skeletal muscle phenotype [11]. Normally, microscopic examination shows tumor cells arranged in sheets and lobules. Cells are pleomorphic, with round to elongated nuclei, and abundant eosinophilic cytoplasm. Areas of necrosis are common. The diagnosis of RMS can be difficult with conventional histological techniques and it could easily be confused with MFH [3, 12]. In such cases immunohistochemistry, electron microscopy, cytogenetics, and DNA flow cytometry are essential for identification of final RMS subtype [12]. Immunohistocytochemistry is necessary to demonstrate cytoplasmic positivity of desmin and nuclear positivity for myogenin. The presence of pleomorphic polygonal rhabdomyoblasts on routine Hematoxylin and eosin stain coupled with immunohistochemical evidence are essential to confirm the diagnosis [6, 12].

Treatment of pleomorphic RMS paratesticular tumour consists mainly of surgical excision and adjuvant chemotherapy and/or radiotherapy. As paratesticular sarcomas are rare there is no standard treatment; however, there is a general consensus that a radical orchiectomy including high ligation of the spermatic cord should be done first [13, 14]. Hemiscrotectomy is performed if initial scrotal incision was made or if the tumour could not be extracted from inguinal orifice [13]. The role of retroperitoneal lymph node dissection (RPLND) is controversial [10, 13, 14] and most authors prefer radiotherapy for the treatment of large lymph node metastasis. The node dissection was not thought to be of therapeutic value but was indicated to stage the disease [10]. Arguments against the routine RPLND are that lymph nodes staging can be made by preoperative imaging studies and that this surgery is associated with significant morbidity. Moreover, microscopic nodal disease can be effectively treated by chemotherapy.

Adjuvant chemotherapy is added after surgery in case of distant metastases to improve prognosis. Many protocols of chemotherapy have been tried. VAC, IVA, and VIE protocols (V: vincristine, A: actinomycin, E: etoposide, I: ifosfamide, and C: cyclophosphamide) were mainly used and better results were observed with VAC protocol [7, 10, 15].

According to Intergroup Rhabdomyosarcoma Study (IRS) classification, patients with metastases at diagnosis are classified as stage IV (14%) (Table 1). They recommend treatment by initial surgical excision followed by chemotherapy with or without radiotherapy [10].

In fact survival rates vary with the initial site and histological subtype of RMS. Pleomorphic RMS is known to have the worst prognosis [3]. Our two patients had unfortunately multiple metastases and lymph node extension at initial diagnosis. Few months after radical orchiectomy they died before or just after starting adjuvant treatment. We believe that pleomorphic RMS has a poor prognosis but patients with initial metastasis should perhaps be managed differently. Recently, Kishore et al. reported histological diagnosis of pleomorphic paratesticular RMS with fine needle aspiration. Patients that have metastatic paratesticular tumours should probably undergo initial biopsy prior to surgical excision and a neoadjuvant chemotherapy and/or radiotherapy should be tried in case of type IV pleomorphic RMS.

## 5. Conclusion

Paratesticular pleomorphic RMS is a rare and aggressive tumour. The diagnosis is made after histological examination. Immunohistochemical and ultrastructural findings could be necessary in some cases. Retroperitoneal lymph node dissection causes a big morbidity and is not recommended. Computed tomography is effective in staging the disease.

The first step of treatment of paratesticular RMS consists of a high inguinal orchidectomy. Adjuvant chemotherapy should be added, with or without radiotherapy, for distant metastases diseases.

Metastatic paratesticular pleomorphic RMS has a poor prognosis.

## Figures and Tables

**Table 1:** IRSG Presurgical Staging Classification

Stage	Sites	Tumor (T)	Size	Node (N)	Metastases(M)
I	Orbit, head and neck (excluding parameningeal) GU: nonbladder/nonprostate	T_1_ or T_2_	a or b	N_0_, N_1_, or N_x_	M_0_
II	Bladder/prostate, extremity, cranial, parameningeal, other (includes trunk, retroperitoneum, and so on)	T_1_ or T_2_	a	N_0_ or N_x_	M_0_
III	Bladder/prostate, extremity, cranial parameningeal, other (includes trunk, retroperitoneum, and so on)	T_1_ or T_2_	a b	N_1_ N_0_, N_1_, or N_x_	M_0_
IV	All	T_1_ or T_2_	a or b	N_0_ or N_1_	M_1_

Note. Tumor: T_1_, confined to anatomic site of origin, (a) ≤5 cm in diameter in size, (b) >5 cm in diameter in size; T_2_, extension and/or fixative to surrounding tissue, (a) ≤5 cm in diameter in size, (b) >5 cm in diameter in size; regional nodes: N_0_, regional nodes not clinically involved; N_1_, regional nodes clinically involved by neoplasm; N_x_, clinical status of regional nodes unknown; metastasis: M_0_, no distant metastasis; M_1_, metastasis present.

Abbreviation: GU: genitourinary.
